# The GIMEMA-ALLIANCE Digital Health Platform for Patients With Hematologic Malignancies in the COVID-19 Pandemic and Postpandemic Era: Protocol for a Multicenter, Prospective, Observational Study

**DOI:** 10.2196/25271

**Published:** 2021-06-01

**Authors:** Fabio Efficace, Massimo Breccia, Paola Fazi, Francesco Cottone, Bernhard Holzner, Marco Vignetti

**Affiliations:** 1 Data Center and Health Outcomes Research Unit Italian Group for Adult Haematologic Diseases (GIMEMA) Rome Italy; 2 Department of Precision and Translational Medicine Sapienza University Rome Italy; 3 University Hospital of Psychiatry II Medical University of Innsbruck Innsbruck Austria; 4 Evaluation Software Development Ltd Innsbruck Austria

**Keywords:** digital health, hematology, leukemia, lymphoma, multiple myeloma, patient-reported outcomes, quality of life, symptoms, COVID-19

## Abstract

**Background:**

The COVID-19 pandemic has raised unprecedented challenges in the management of patients with cancer and has increased the demands for digital health tools that, for example, could facilitate remote monitoring of patients. Based on this, the Gruppo Italiano Malattie Ematologiche dell’Adulto (GIMEMA) has recently developed a digital health tool dedicated to patients with hematologic malignancies: the GIMEMA-ALLIANCE platform.

**Objective:**

The main objectives of this web-based platform are to generate relevant data to better understand quality of life, symptoms, and medication adherence during the COVID-19 pandemic and postpandemic era; to develop a prospective real-life registry on outcomes of patients with hematologic cancer, with or without a diagnosis of COVID-19; and to facilitate patient-centered care in routine practice.

**Methods:**

The platform consists of physician- and patient-secure portals and enables electronic patient-reported outcome (ePRO) assessments with real-time graphical presentation to physicians of individual patient symptoms and quality-of-life outcomes. Automated alerts are sent to treating hematologists based on the following predetermined criteria: presence of clinically important problems and symptoms, problems with adherence to therapy, and risk of COVID-19 diagnosis. The platform also allows physicians to set up video consultations. Clinical information regarding disease and treatment as well as clinical and survival outcomes are also prospectively collected.

**Results:**

Recruitment of participants started in December 2020. As of April 2021, a total of 116 patients have been enrolled in this study. Use of this platform may help to improve patient-physician communication and help hematologists in the early recognition of clinically important problems and symptoms of their patients. More than 20 community and university-based hospitals have currently agreed to participate. In addition to patient-reported outcome data, the prospective collection of disease- and treatment-related information, as well as data on possible COVID-19 diagnosis and COVID-19 vaccination, will allow the development of a large database to also identify subgroups of patients at risk of poor outcomes.

**Conclusions:**

Data generated via this platform will help to answer clinically relevant questions for patients with hematologic malignancies during the COVID-19 pandemic and postpandemic era. The use of the GIMEMA-ALLIANCE platform in routine practice may also contribute to enhancing patient-centered care.

**Trial Registration:**

ClinicalTrials.gov NCT04581187; https://clinicaltrials.gov/ct2/show/NCT04581187

**International Registered Report Identifier (IRRID):**

PRR1-10.2196/25271

## Introduction

### Background

The COVID-19 pandemic has recently transformed health care delivery all over the world by also remarkably increasing the complexity of clinical decision making in oncology [1].

Approaches to obviate the need for physical meeting between patients and health care providers (ie, telephone calls, communication by mail, and mobile phone apps) are now more frequently preferred to limit the risk of virus spread among uninfected patients [2]. A rapid integration of digital health technologies in the health care system is being embraced in several countries to achieve a new balance between self-isolation and health care delivery [3,4]. Addressing the physical and mental health-related concerns of patients is now critical [5] and may have major implications. For example, empirical data indicate that patients’ anxiety related to the COVID-19 pandemic may be associated with the risk of underestimating important symptoms of hematologic malignancies, thereby delaying diagnosis and appropriate treatment [6].

The COVID-19 emergency has prompted new questions about how to integrate the vulnerability of patients with cancer—because of either the disease itself, the effects of cancer treatments, or additional comorbidities—with the risk of COVID-19 [7].

Management of patients with hematologic malignancies during the COVID-19 pandemic can be particularly challenging for several reasons, including the need for expert supportive care services, which can be substantially limited during the current clinical scenario. For example, careful evaluation of how long patients with leukemia can be managed without in-person follow-up visits, blood tests, and therapies, as well as close monitoring of potential complications during therapy, are now critical components of patient management during this global pandemic [8]. Therefore, special recommendations to optimize treatments for patients with hematologic malignancies during the COVID-19 pandemic have been recently published [8]. A recent multicenter study, which included a large sample of more than 500 adult patients with hematologic malignancies who required hospitalization for COVID-19, also indicated high mortality rates [9]. This study showed that patients with hematologic malignancies with COVID-19 had worse outcomes than both the general population with COVID-19 and patients with hematologic malignancies without COVID-19 [9].

### The Added Value of Patient-Reported Outcome Assessment in Routine Cancer Care

There is now convincing evidence of the clinical value of systematic collection and use of patient-reported outcomes (PROs) in routine cancer practice [10,11]. Also, empirical data indicating that PROs, including symptoms and functional aspects, provide independent prognostic data for survival [12,13] further reinforces the need to more systematically assess PROs, as they provide unique information that cannot be captured via traditional clinical measures or laboratory exams.

Regular assessment of PROs in daily practice has been shown to provide a number of positive effects with regard to, for example, improved symptom control, timely identification of physical and psychosocial needs, improved patient-physician communication, and improved health-related quality of life (HRQoL) and emotional well-being, without increasing patient management activities for health care professionals [10,11,14-21].

Digital health technology now allows implementation of electronic PRO (ePRO) measures that patients can complete, for example, remotely via web platforms. Results obtained by completing ePROs via these platforms are typically graphically displayed in real time and available for physicians and clinical staff.

Notably, one randomized controlled trial (RCT) conducted in patients with advanced solid cancer reported that web-based self-reporting of symptoms with automated alerts to clinicians for severe or worsening symptoms was not only associated with a reduced number of emergency room visits and hospitalizations but also with significant improvement in overall survival [14,22]. Similarly, another RCT in patients with advanced-stage lung cancer showed that a web-mediated follow-up algorithm based on self-reported symptoms also improved overall survival [23]. A more recent RCT conducted in patients with solid cancer, who were mainly treated with curative intent, showed that real-time monitoring with ePROs improved physical well-being and self-efficacy without increasing hospital workload, and authors concluded that online symptom monitoring is a feasible approach to be implemented in routine care [24].

The key advantages of using ePROs during the COVID-19 pandemic have been recently highlighted [25] and these may include the following: the prevention of the occurrence of severe adverse events and the prompt management of medical needs. While some challenges exist to the implementation of ePRO instruments into routine practice, such as technical and organizational factors, a number of actions can be put in place to maximize efficiency and ensure successful collection of ePROs [26].

### Study Rationale and Hypotheses

Italy was one of the most severely affected countries by the COVID-19 pandemic in early 2020 [27], putting enormous pressure on hematology departments across the country.

The current pandemic represents an opportunity to further boost use of digital health technologies in the hematology arena and maximize the use of remote ePRO data collection in patients with hematologic malignancies. As recently observed, the COVID-19 pandemic has precipitated a shift to remote technology-enabled care [24].

In this new clinical scenario, the Gruppo Italiano Malattie Ematologiche dell’Adulto (GIMEMA) led the development of a dedicated digital health tool for patients with hematologic malignancies. The tool was called *An online platform to improve patient-centered care during the COVID-19 pandemic: A GIMEMA surveillance program in hematologic malignancies* (ALLIANCE)—the GIMEMA-ALLIANCE platform.

GIMEMA is a nonprofit research organization with a long-standing history of clinical research in hematology; it consists of a well-established network of about 150 affiliated hematology centers throughout Italy, including both community and university-based hospitals, plus a data center located in Rome that coordinates research activities. The GIMEMA-ALLIANCE platform was devised in collaboration with Evaluation Software Development (ESD), a well-established spin-off digital health company, whose key product is the Computer-based Health Evaluation System (CHES) infrastructure. CHES is a tool for the electronic collection, calculation, analyses, and presentation of PROs. CHES is used worldwide in more than 75 hospitals and research institutions for the electronic capture of medical and PRO data [28,29].

We hypothesize that use of the GIMEMA-ALLIANCE platform in patients with hematologic malignancies would facilitate a more patient-centered care approach and would support hematologists in the earlier recognition of clinically important problems and symptoms of their patients. By using this platform, we also aimed to collect disease- and treatment-related information, as well as prospective PRO data, to answer important research questions.

### Objectives

The main objectives of the GIMEMA-ALLIANCE platform are as follows:

1. To generate relevant data to better understand quality of life, symptoms, and medication adherence in adult patients with hematologic malignancies during the COVID-19 pandemic and postpandemic era, and to identify subgroups of patients at higher risk of poor outcomes.
2. To develop a large prospective real-life registry on cancer treatment outcomes of patients with hematologic cancer, with or without a diagnosis of COVID-19.
3. To facilitate patient-centered care in routine practice by using ePRO measures with automated alerts sent to treating hematologists.

## Methods

### Study Participants

Considering that we aimed to use the platform with patients seen in daily practice, very broad inclusion criteria were set up. Inclusion criteria were as follows: (1) diagnosis of any hematologic malignancy according to the 2016 World Health Organization classification [30], (2) adult patients (≥18 years of age), and (3) written informed consent. Exclusion criteria included (1) major cognitive deficits or psychiatric problems hampering a self-reported evaluation and (2) inability to read and understand the local language. Patients enrolled in clinical trials are still eligible for this protocol, and this information is recorded in the case report forms (CRFs) at the time of entry in this study to be used for sensitivity analyses.

### Ethics Approval, Consent to Participate, and Trial Registration

This study is currently funded by GIMEMA and was approved by the Ethics Committee of the University of Sapienza, Rome (study reference No. 5822, Protocol 366/2020). Documented informed consent must be obtained for all patients. By means of the information sheet, all eligible patients will be informed about the aims of the project, the procedures to participate in the project, and the strict confidentiality of the patients’ data. It will be emphasized that participation is voluntary and that it might contribute to defining a more personalized and timely therapeutic approach. It will be also highlighted that either refusing to join the study or withdrawing afterwards at any time will not prejudice the patient’s subsequent care. This trial was registered at ClinicalTrials.gov (NCT04581187).

### Patient Recruitment Procedures

After approval by the local Ethics Committee of each participating center, patients who meet the inclusion criteria will be invited to participate by authorized investigators. At present, 27 centers have agreed to participate. Eligible patients willing to participate will be given a written information sheet and asked to sign the informed consent form. Considering the challenges posed by the pandemic, with inevitably less frequent face-to-face access in the hospital, and in line with current Italian regulatory guidelines, consent may also be anticipated, for example, via telephone or email [31].

All eligible and consenting patients will be registered in the platform via the dedicated physician portal, which allows for the downloading of patient log-in details and basic instructions to access the patient portal, to be handed over to patients.

### Study Design and Logistics

This is a multicenter prospective observational study led by GIMEMA. All data will be centrally collected and analyzed at the GIMEMA Data Center, which operates according to high-quality and transparent standards of clinical research, as certified by the European Clinical Research Infrastructure Network (ECRIN) [32]. ECRIN certification ensures that a data center meets several procedural standards with respect to different aspects of clinical research. These include the design, development, and validation of CRFs; data entry and data management; coding of variables; validation of databases; and statistical analysis. ECRIN certification also concerns the management of information technology infrastructure; data security, storage, and access; and back-up procedures.

The platform will be open for 2 years to register new patients who will be followed up for 2 years from the date of registration; the platform consists of two password-protected secure portals, one for physicians [33] and one for patients [34]. Data security of the web-collection system in this study is fully compliant with the highest standards of security of data protection (see details in the next paragraph).

Once a participating center obtains local ethical approval and it is officially ready (ie, having sorted out all administrative steps to receive access to the platform by the GIMEMA Data Center), a training session is organized by the GIMEMA-ALLIANCE management team with the on-site clinical staff of the participating hospital. The session broadly aims at instructing the local staff in using the platform and in interpreting patient-reported data. A brief practical guide is also sent to the clinical staff at study start-up. A study newsletter is circulated on a regular basis to provide feedback on study progress and to invite users to report any questions or requests of assistance to the GIMEMA-ALLIANCE management team at their email address.

### The Architecture of the GIMEMA-ALLIANCE Platform

**Figure 1:**
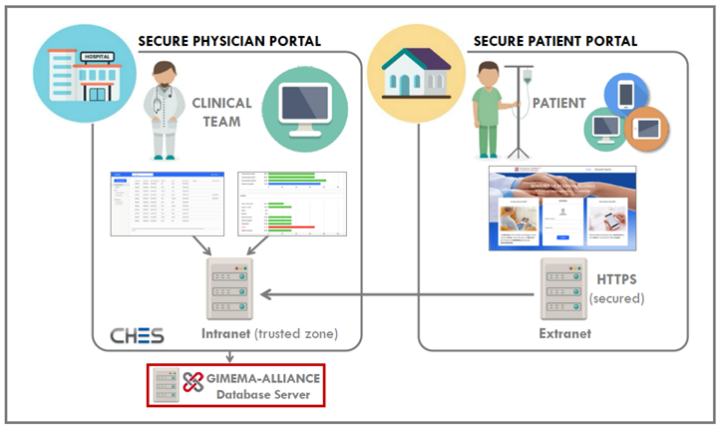
Main workflow of the GIMEMA-ALLIANCE platform. ALLIANCE: An online platform to improve patient-centered care during the COVID-19 pandemic: A GIMEMA surveillance program in hematologic malignancies; CHES: Computer-based Health Evaluation System; GIMEMA: Gruppo Italiano Malattie Ematologiche dell’Adulto.

All clinical and patient-reported data in this study will be collected through the dedicated web platform using the CHES infrastructure [28]. CHES is a sophisticated web-based software tool specifically designed for ePRO questionnaire administration, real-time graphical presentation of individual patient results, and clinical and PRO data collection, storage, and analysis. This platform runs in all common web browsers, devices, and operating systems; it has already been implemented in a number of national and international institutions engaging clinical studies with electronic capture of medical and PRO data as well as PRO data collection in routine oncology care [35-40]. Since CHES is a web-based application, the communication between the client (ie, browser) and the server needs to be protected from being read or modified by an attacker. This is done by only allowing Secure Sockets Layer–encrypted communication (ie, https). The https encrypts the data between the client and the server, so no other person can read or modify the data sent from the client to the server and from the server to the client. These secure web links are used for all participants within the CHES system: administrators, physicians, and patients. To further ensure data security, the system is divided into two different zones, the so-called trusted and untrusted zones. These zones are realized by two different servers that differ in their accessibility. The server in the trusted zone is only accessible within the GIMEMA network, whereas the server in the untrusted zone is accessible via the World Wide Web. A network connection exists only from the trusted zone to the untrusted zone but not vice versa. All sensitive patient data are stored in the trusted zone. Only data that cannot be linked to a patient are stored in the untrusted zone. To fetch data from the untrusted zone (eg, the completed survey), a software service runs in the trusted zone that merges the data from the untrusted zone into the database of the trusted zone. Only the trusted zone knows the mapping of the anonymous patient ID from the untrusted zone to the actual patient ID in the trusted zone (Figure 1).

For accessing the system, two different weblinks exist. One weblink is used to access the patient portal in the untrusted zone, whereas the other weblink is used to access the physician portal in the trusted zone. Any system access requires at least a username and a password. Furthermore, for accessing the physician portal, a two-factor authentication mechanism is in place that requires each physician to enter a 6-digit code that is sent to his or her mobile phone when trying to log in. This token is valid only once and expires after 3 minutes if not used. The credentials for the physician portal will be provided by the GIMEMA-ALLIANCE management team to all investigators, who have been previously authorized to access the physician portal, prior to the study start date at each participating center. The credentials to the patient portal will be provided by the local investigators to the patients enrolled at their participating center. The physician web interface is designed for an easy-to-use approach, allowing quick access to all of their patients’ relevant data and real-time access to a visual summary of patient-reported information via the web browser of each patient enrolled within the same hospital. The physicians will hand out a flyer to their patients containing the individual credentials, along with the weblink to access the patient portal.

**Figure 2:**
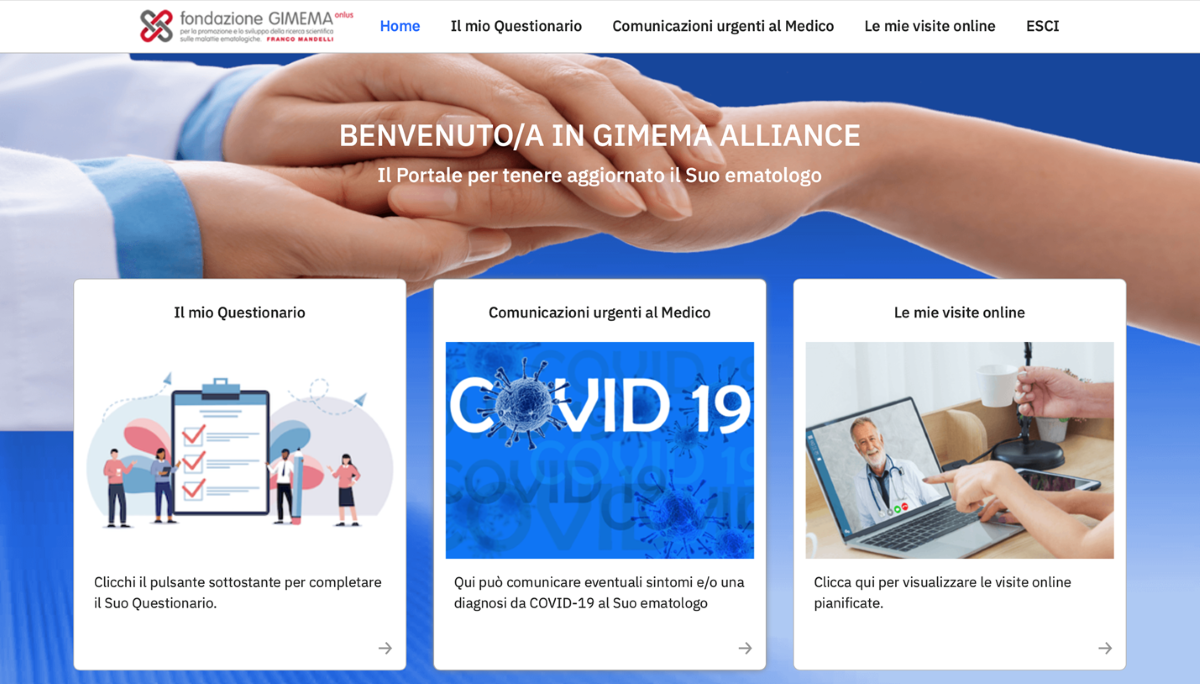
GIMEMA-ALLIANCE patient portal. ALLIANCE: An online platform to improve patient-centered care during the COVID-19 pandemic: A GIMEMA surveillance program in hematologic malignancies; GIMEMA: Gruppo Italiano Malattie Ematologiche dell’Adulto.

Once the patient is entered into the dedicated portal, three modules are displayed (Figure 2). The first module mainly aims at collecting patient-reported information on quality of life, symptoms, and medication adherence (ie, the ALLIANCE survey, which is described in detail in the next section). The second module can be used by patients to provide real-time information to their treating hematologists regarding a potential risk of SARS-CoV-2 infection or a confirmed COVID-19 diagnosis. Items included in this module are as follows: having received a confirmed diagnosis of COVID-19, having been in close contact with someone with a COVID-19 diagnosis, having reported a fever of more than 37.5 °C, and having experienced breathing difficulty, cough, or an altered sense of smell and/or taste over the last 2 weeks. The third module consists of the video consultation area, which allows patients to have online visits with their physicians and allows the actual scheduling of online visits via a user-friendly agenda.

### The ALLIANCE Survey

The first module of the patient portal includes the ALLIANCE survey, which aims to assess the patient’s perspective on the following broad areas: global health status and quality of life, functional aspects, symptoms, and problems with adherence to therapy. In addition, a few information items (eg, questions about social support) are only included in the first survey. From the second assessment on, these few additional information items will no longer be requested of patients.

Just after registration in the study, patients will be asked by their treating hematologist to complete the survey at their earliest convenience. Considering the real-life nature of the study, and the wide range of clinical scenarios that could be represented in this platform, no prespecified time points for completion have been preplanned. In any case, the platform is designed to send regular reminders to patients to complete the survey, with the first one being sent after 1 week from date of registration, that is, if the first survey has not been completed within the first week of registration.

Global health status and quality of life, functional aspects, and symptoms are evaluated with the European Organisation for Research and Treatment of Cancer (EORTC) Quality of Life Questionnaire–Core (QLQ-C30) and ad hoc items from the EORTC Item Library. The EORTC QLQ-C30 is a brief, multidimensional HRQoL measure consisting of 30 items; the measure includes five functional scales (ie, physical, role, emotional, social, and cognitive), three symptoms (ie, fatigue, nausea and vomiting, and pain), a global health status and quality of life scale, and six single items (ie, dyspnea, insomnia, appetite loss, constipation, diarrhea, and financial difficulties) [41]. To further increase the sensitivity of HRQoL measurement for patients with hematologic malignancies, four ad hoc items from the EORTC Item Library are also included [42].

The decision on items to be included in the survey from the EORTC Item Library was based on clinical grounds after discussion with physicians involved in the project, on which symptoms not already covered by the EORTC QLQ-C30 would be most relevant for the majority of patients with hematologic malignancies. Also, we limited the inclusion to only four additional symptoms to reduce response burden as much as possible. Specifically, two items stemmed from the EORTC Quality of Life Questionnaire–Chronic Lymphocytic Leukemia (QLQ-CLL17) module (ie, night sweats and bone pain), and two items stemmed from the EORTC Quality of Life Questionnaire–Chronic Myeloid Leukemia (QLQ-CML24) module (ie, headache and muscular cramps).

We used the EORTC QLQ-C30 questionnaire, as it covers not only functional aspects, global health status, and HRQoL of patients, but it also includes a core set of cancer symptoms that we considered important to monitor in our patients. This questionnaire has been used in a number of studies of patients with hematologic malignancies [43], and its measurement invariance in the setting of hematology has been supported [44]. In addition, the EORTC QLQ-C30 also allows the calculation of a single summary score, which has been validated across the whole spectrum of both solid and hematologic malignancies [45,46] and may be helpful in future analyses with data collected in our platform. Indeed, recent empirical data in the real-world setting, for example, have shown that this EORTC QLQ-C30 summary score has a strong prognostic value for overall survival across a wide range of cancer populations above and beyond that provided by traditional clinical and sociodemographic variables [12]. Furthermore, international general population normative data are available for this questionnaire [47], thereby facilitating future comparisons of the profiles of our patients with reference values. Finally, the availability of recently established evidence-based criteria for the definition of clinically important problems and symptoms that can be used to ease interpretation of data in daily clinical practice [35] is an additional key strength we valued highly for the inclusion of the EORTC QLQ-C30 in our platform. Indeed, as previously observed, one of the key challenges to the successful implementation of PRO data with health information technologies for use in routine care is clinician understanding of how to interpret and respond to PRO information [48].

Medication adherence is evaluated with the Adherence to Refills and Medications Scale (ARMS). We used the shortened 7-item version of the ARMS (ie, ARMS-7), which is a brief self-reported validated measure of medication adherence and correlates very highly with the full 12-item measure [49,50]. Each of the items allows patients to express, on a 4-point scale, how often they do not take or refill their medications under different circumstances. Scores on the ARMS-7 range from 7 to 28, with lower scores indicating better adherence; in addition, the scale is available in Italian.

Adherence to oral anticancer therapies is a known problem, and several factors have been found to be associated with poor medication-taking behaviors, including both treatment-related aspects and personal patient factors [51]. Considering the potential negative effects of suboptimal adherence on clinical efficacy, and the fact that many patients with hematologic malignancies are now treated with oral drugs, we aim to also include in the platform a brief and easy-to-use self-reported measure to assess medication adherence. Among the available self-report adherence measures that can be used in clinical practice [52], we selected the ARMS, which has been shown to be a valid and reliable scale, with optimal performance characteristics among patients with low literacy as well [49]. We considered this latter characteristic of critical importance for inclusion in our platform, considering that we aimed to capture this type of data in real life, hence, approaching patients with various levels of education.

### Active Monitoring of Patients Via the GIMEMA-ALLIANCE Platform

Currently, the platform triggers automated email alerts to the treating hematologist based on the following criteria:

1. Presence of clinically important problems and symptoms.
2. Problems with adherence to therapy.
3. Risk of COVID-19 diagnosis.

**Figure 3:**
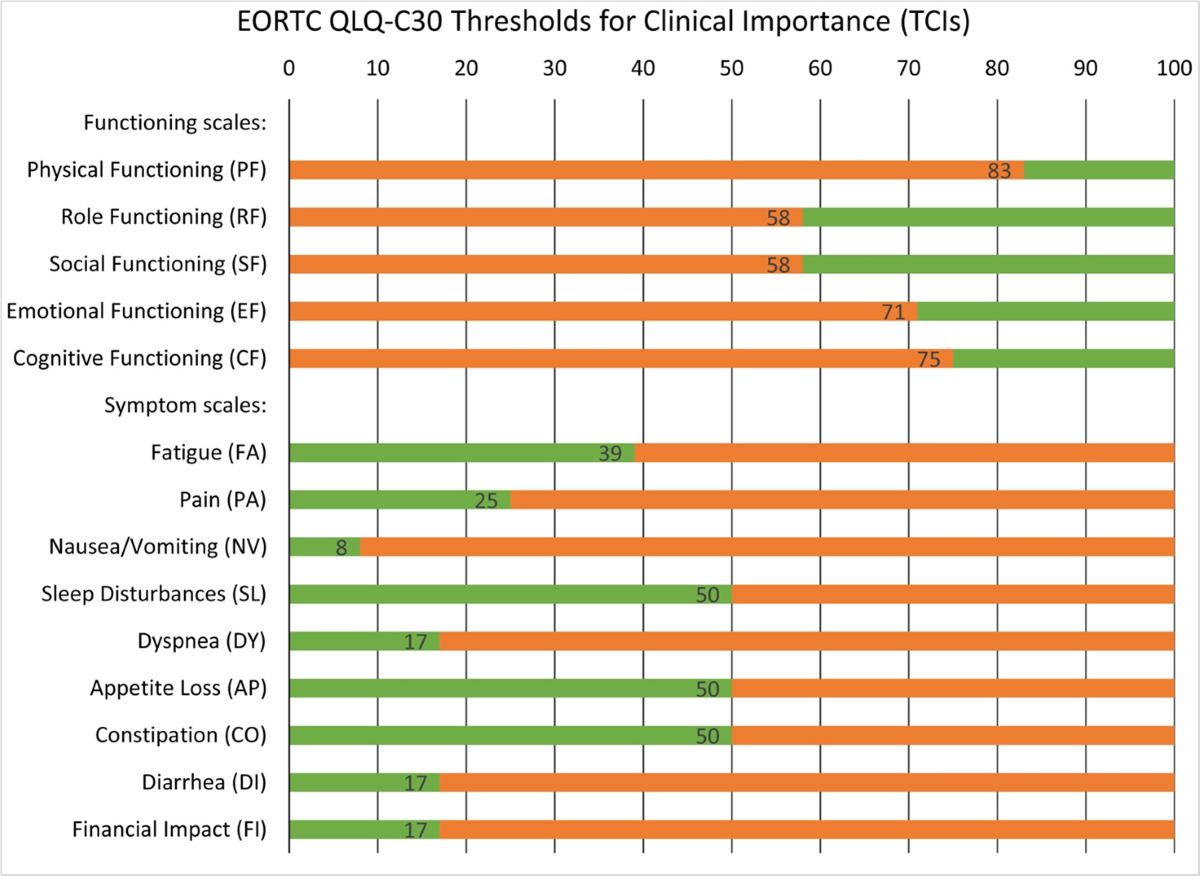
EORTC QLQ-C30 thresholds for clinical importance (TCIs). TCIs are shown inside the bars. Patient scores in the orange range of the bar (ie, below the TCI for functioning scales or above the TCI for symptom scales) indicate clinically important problems or symptoms. Reprinted from Giesinger et al (2020) [35]). EORTC: European Organisation for Research and Treatment of Cancer; QLQ-C30: Quality of Life Questionnaire–Core.

The definition of *clinically important* problems and symptoms is based on previously defined thresholds for functional aspects (eg, physical and emotional functioning) and key cancer symptoms from the EORTC QLQ-C30 [35]. These thresholds for clinical importance were established to detect health problems that limit a patient’s daily life, cause worry to the patient and/or to his or her partner or family, or require help or care; all of these are criteria that have previously been identified as making a health problem relevant for the clinical encounter [35]. The scale-specific thresholds used are reported in Figure 3.

**Figure 4:**
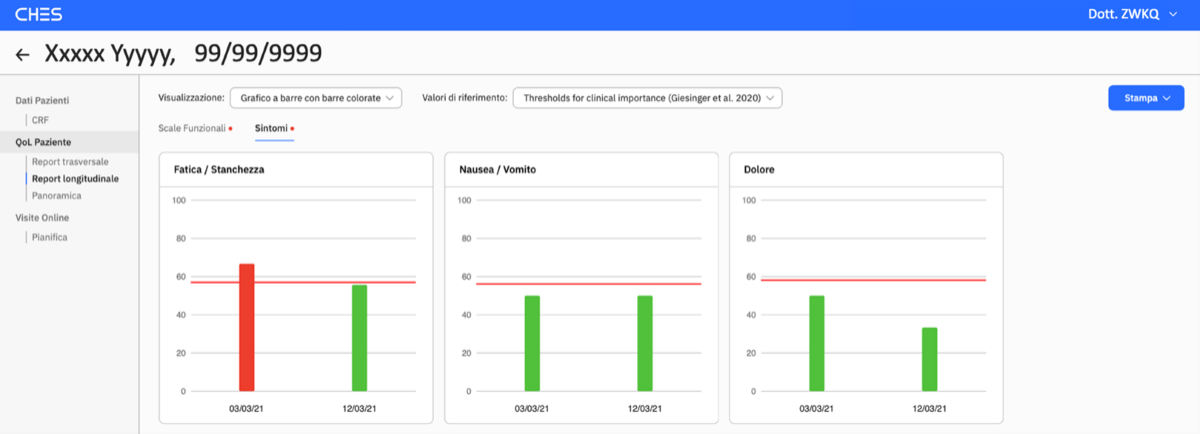
Example of graphical display of results of patient-reported symptoms available on the physician portal. The red vertical bar indicates a clinically important symptom.

Through their portal, physicians have real-time access to a visual summary of patient-reported information, with red bars flagging clinically important problems and symptoms. To further facilitate the timely recognition of potential problems of their patients, email alerts sent to physicians also contains a link directly connecting to the graphical summary generated by the patient ratings. Figure 4 depicts an example of a graphical display of patient-reported symptoms.

Our working definition of potential problems with adherence to therapy is based on the scoring instructions for the ARMS-7 questionnaire, which is used in the platform to assess medication adherence. The overall score from this questionnaire ranges from 7 (best adherence) to 28 (worst adherence) [49,53], and it can be dichotomized as 7 or >7. A score equal to 7 indicates an optimal adherence, while any score larger than 7 indicates some degree of nonadherence. Therefore, we designed the platform to send an alert for adherence if the total score from ARMS-7 is higher than 7.

Based on patients’ answers to items included in the *risk of COVID-19 diagnosis* module, the platform automatically generates real-time alerts if at least one of them is checked to be true. This alerting procedure aims to enhance physician ability to more proactively engage patients who are at heightened risk of being diagnosed with COVID-19 or who may have been actually diagnosed with the disease.

However, considering the real-life nature of this study involving several centers, the alerting algorithm currently implemented for triggering alerts could be further refined or revised during the study period. Such information will be recorded in study files.

**Figure 5:**
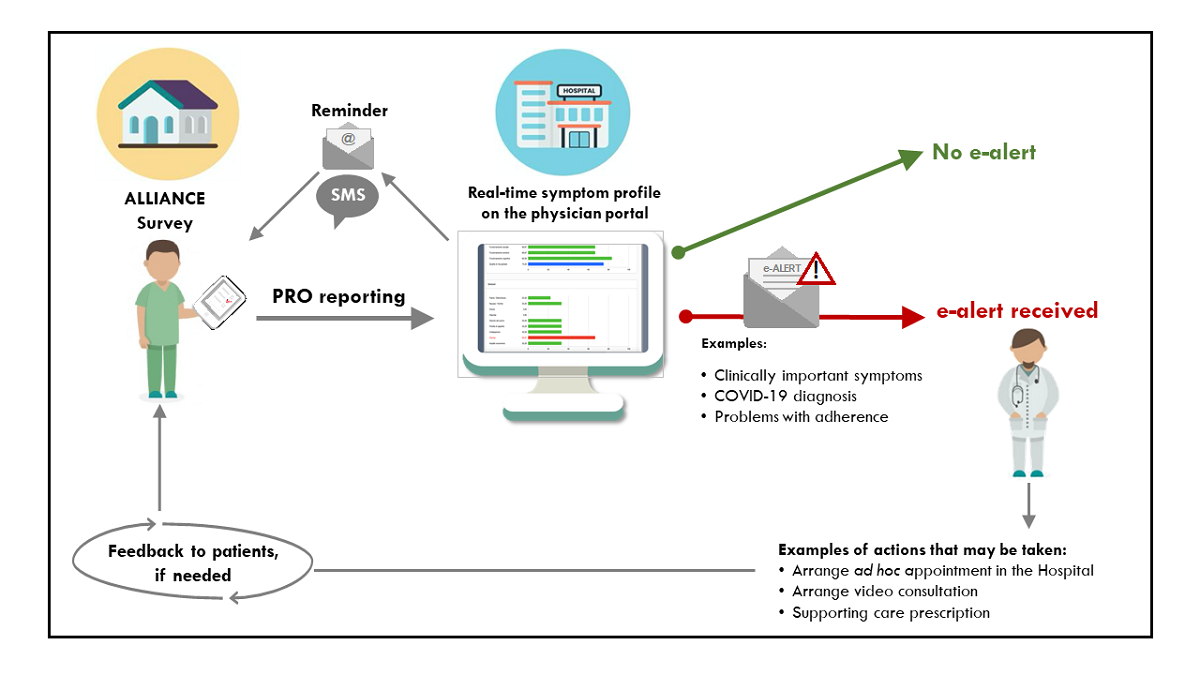
Schematic workflow of the patient-generated alerts to the medical team. ALLIANCE: An online platform to improve patient-centered care during the COVID-19 pandemic: A GIMEMA surveillance program in hematologic malignancies; PRO: patient-reported outcome.

Physicians are free to decide on which action they feel to be most appropriate for their patients. For example, depending on the type and/or frequency of patient-generated alerts, they could (1) arrange an ad hoc face-to-face visit in the hospital, (2) arrange a video consultation, (3) refer the patient to other specialists, or (4) simply contact the patient by phone to further understand the patient’s needs. The workflow of the alerting procedure is summarized in Figure 5.

Automated alerts are only meant to help physicians in the early recognition of potential problems of their patients; therefore, predetermined actions in response to specific alerts are not suggested by the platform. Considering the large heterogeneity of patient characteristics and types of specific treatments patients may receive, we did not recommend specific actions to take in response to alerts.

In any case, information about actions taken by physicians will be collected with ad hoc surveys sent to them, to understand how the platform might have helped them in the clinical management of their patients. These surveys will include questions about actions taken after having received an alert, such as *no action taken*, *phone call with the patient* to further understand the patient’s needs, *video consultation*, or having arranged a *visit in the hospital* or *referral to other specialists*. In addition, the survey will ask the physician about the frequency of his or her access to the platform and will also include 14 questions about acceptability and feasibility of the portal; responses could include, for example, “Use of the platform improved my understanding of the actual patient burden of symptoms” or “I think that the platform might be improved by additional features.”

### Physician-Reported Information

The physician portal also includes a set of CRFs that are to be completed by the treating hematologists at baseline (ie, at study entry) and at regular intervals. Also, ad hoc CRFs are to be completed regarding COVID-19 diagnosis and COVID-19 vaccination, if applicable. Table 1 provides a summary of CRFs currently uploaded in the physician portal.

The baseline form will require a number of sociodemographic as well as disease- and treatment-related variables. Some key information items collected in this form are provided Textbox 1.

**Table 1:** Summary and timeline of case report form (CRF) completion by the investigator.

CRFs to be completed	Frequency of completion	Timeline	Purpose
Registration checklist	Once	At the time of registration in the platform	To confirm eligibility criteria
Baseline form	Once	At the time of study entry	To comprehensively understand patients’ characteristics and disease status at study entry
Follow-up form	On a regular basis	Every 3 months	To monitor treatment, disease progression, and status of patient (including survival status) at follow-up
COVID-19 form	As appropriate	Only in the case of confirmed diagnosis with COVID-19	To collect information on COVID-19 severity, symptoms, and outcomes
COVID-19 vaccine form	As appropriate	Only in the case that patients receive a COVID-19 vaccine	To better understand the implications of having received a vaccine

**The main clinical and sociodemographic variables included in the baseline case report form to be completed at study entry.**

Selected key variables:

- Type of hematologic malignancy
- Date of initial diagnosis
- Patient date of birth
- Sex
- Education level
- Smoking status
- Type and number of comorbidities
- Eastern Cooperative Oncology Group performance status
- Status of patient at study entry: options include newly diagnosed and yet untreated, initial diagnosis and receiving first-line treatment, in remission and not receiving treatment, in remission and receiving consolidation or maintenance treatment, stable but not in remission, and relapsed or refractory
- Ongoing treatment (if applicable)

The follow-up form will be completed every 3 months and will require basic data about ongoing therapy, any major toxicity, response to therapy, as well as survival status. In addition, the COVID-19 form, which will be completed only if the patient receives a diagnosis of COVID-19, will require specific information related to COVID-19 occurrence and related treatment history, hospitalization, symptoms, and outcomes, including survival. Similarly, a COVID-19 vaccination form is available to be completed, as appropriate, to collect information on date and type of vaccine received.

However, given the evolving situation of the COVID-19 pandemic, additional information regarding the patient’s medical history or clinical course of the disease (eg, details on disease characteristics, treatments, and possible complications) may be further requested of investigators, who may be asked to answer specific research questions of clinical relevance.

### Statistical Analysis

Given the nature of this study, a formal calculation of sample size and power was not performed. However, at the time of study design, we determined that 400 patients in 2 years was a reasonable estimate of the overall enrollment of this study. Each patient enrolled in this study will be followed up for 2 years from the date of registration. All the analyses described below will be performed overall and by patient subgroups (eg, either by type of hematologic disease at study entry, type of treatment, or diagnosis of COVID-19). We will estimate the trajectories over time of the scores from the scales from all PRO questionnaires by either a generalized linear mixed model (GLMM) or a growth curve model [54], depending on the actual observed timing of PRO assessments, to prospectively monitor HRQoL, symptoms, and adherence to therapy. We will also assess the prevalence over time of clinically important problems and symptoms, as measured by the EORTC QLQ-C30, using the GLMM. For each scale, the clinical relevance of HRQoL problems will be determined according to previously published thresholds [35]. The GLMM approach will be used to investigate factors associated with physical and mental health concerns and adherence to therapy. Depending on the type of outcome to be investigated, we will use either the GLMM, the Cox proportional hazards model, or the Fine-Gray model for competing risks [55] to assess the impact on patient outcomes (eg, survival outcomes) of key factors, including the diagnosis of COVID-19.

The clinical strategies adopted by physicians in response to patient-generated alerts, as well as patient characteristics and information about disease course and outcomes, will be summarized using means, proportions, medians, and interquartile ranges, according to the type of variable. Possible cross-sectional comparisons of outcomes between subgroups of patients, based on, for example, type of hematologic disease, type of treatment, or diagnosis of COVID-19, will be assessed using either Fisher exact, chi-square, Wilcoxon-Mann-Whitney, or Kruskal-Wallis tests, according to the type of variable.

## Results

Recruitment of participants started in December 2020. As of April 2021, a total of 116 patients have been enrolled in this study. The main outcomes from this project will include longitudinal patterns of patients’ self-reported health issues and needs related to their overall quality of life, symptoms, and medication adherence. In addition, the real-time flow of information between patients and their physicians will possibly improve communication and help physicians in adopting more timely interventions. Data accumulated via this platform may also lay the groundwork to better understand the implications of a COVID-19 diagnosis on the midterm to long-term mental and physical health of patients with hematologic malignancies.

All findings will be disseminated in international peer-reviewed journals and abstracts presented at major international conferences. The study coordinators and the GIMEMA Data Center must approve all publications, abstracts, and presentations based on patients included in this study. Given the paucity of information on the effects of COVID-19 in patients with cancer and the importance of promptly providing new information to the scientific community, as well as in the best interest of patients, interim analyses and publications are foreseen during the recruitment period.

## Discussion

We expect this platform to eventually become a large database containing information on the clinical course of the disease, patient-reported quality of life, and symptom profiles of patients with hematologic malignancies. Data collected in this platform will also allow us to compare the outcomes of patients with hematologic malignancies who may have been diagnosed with COVID-19 with those without COVID-19. Indeed, there is evidence indicating that the COVID-19 pandemic poses major risks to patients with hematologic malignancies [9].

We also note that this new platform, whose development was prompted by the global pandemic, can be an opportunity to further boost a shift toward a more patient-centered healthcare paradigm in the hematology arena. An essential aspect of the platform is that of being based on PROs, hence, making the patient’s input a critical aspect for its functioning.

This platform has been devised to be used across all hematologic malignancies and can also be customized with additional specific modules and functionalities, for example, to address the needs of specific hematologic populations or to address specific research questions. The GIMEMA-ALLIANCE infrastructure may also be used for the conduct of multiple studies. Finally, having been purposely devised as a multilingual web-based tool, it can be easily used in international contexts and implemented for use in other countries. Therefore, given the enormous pressure put on health care systems since the surge of the COVID-19 pandemic and the urgent need to implement digital health tools that can facilitate cancer care, we also welcome international collaborators to join our efforts in this area.

## Abbreviations

ALLIANCE: An online platform to improve patient-centered care during the COVID-19 pandemic: A GIMEMA surveillance program in hematologic malignancies
ARMS: Adherence to Refills and Medications Scale
ARMS-7: 7-item version of the Adherence to Refills and Medications Scale
CHES: Computer-based Health Evaluation System
CRF: case report form
ECRIN: European Clinical Research Infrastructure Network
EORTC: European Organisation for Research and Treatment of Cancer
ePRO: electronic patient-reported outcome
ESD: Evaluation Software Development
GIMEMA: Gruppo Italiano Malattie Ematologiche dell’Adulto
GLMM: generalized linear mixed model
HRQoL: health-related quality of life
PRO: patient-reported outcome
QLQ-C30: Quality of Life Questionnaire–Core
QLQ-CLL17: Quality of Life Questionnaire–Chronic Lymphocytic Leukemia
QLQ-CML24: Quality of Life Questionnaire–Chronic Myeloid Leukemia
RCT: randomized controlled trial
